# Polypeptoid Monomer Sequence and Chemical Composition as Independent Controls of Interfacial Tension and Elasticity at Air/Fluid Interfaces

**DOI:** 10.1021/acs.langmuir.4c02195

**Published:** 2024-07-12

**Authors:** Michal Roguski, Michael L. Davidson, Audra J. DeStefano, Rachel A. Segalman, Lynn M. Walker

**Affiliations:** † Department of Chemical Engineering, Carnegie Mellon University, Pittsburgh, Pennsylvania 15213, United States; ‡ Department of Chemical Engineering, University of California, Santa Barbara, California 93106, United States; § Department of Materials, University of California, Santa Barbara, California 93106, United States; ∥ Department of Chemical Engineering & Materials Science, University of Minnesota, Minneapolis, Minnesota 55455, United States

## Abstract

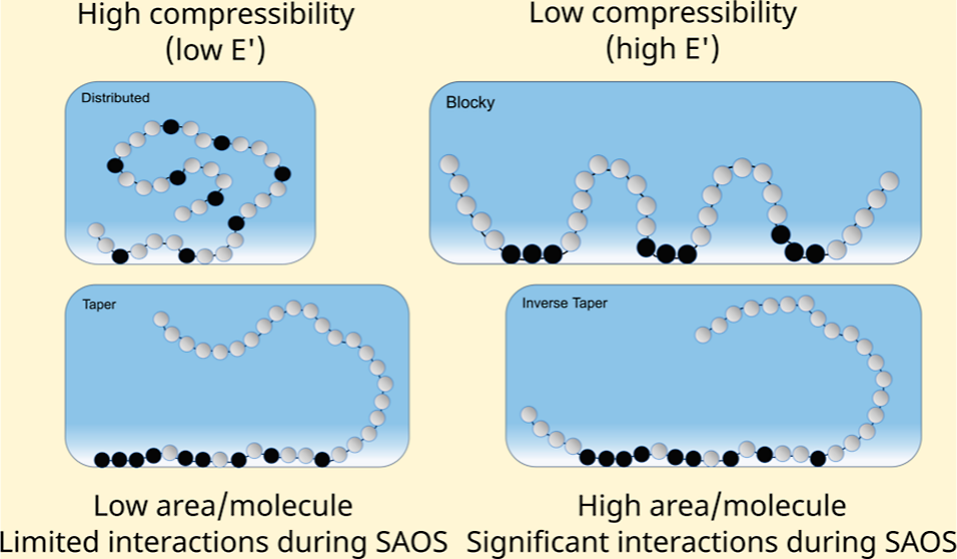

We use sequence-specific polypeptoids to characterize the impact of the monomer sequence on the adsorption of surface-active polymers at fluid/fluid interfaces. Sets of 36 repeat unit polypeptoids with identical chemical composition, but different sequences of hydrophobic moieties along the oligomer chain (taper, inverse taper, blocky, and evenly distributed), are designed and characterized at air/water interfaces. Polypeptoids are driven to the interfaces by decreasing the solvent quality of the aqueous solution. In situ processing of the adsorbed layers causes a collapse of polypeptoids and the formation of irreversibly adsorbed, solvent-avoiding layers at interfaces. Differences in thermodynamic properties, driven by solubility, between the collapsed structures at interfaces are studied with measurements of interfacial tension. The dilatational modulus of polypeptoid-coated interfaces is used as a proxy to probe the extent of the coil–globule collapse at the interface. The role of hydrophobicity is investigated by comparing four sequences of polypeptoids with an increased size of the hydrophilic side chains. In each set of polypeptoids, the composition of molecules, not the sequence, controls the surface concentration. The molecules are described in terms of the distribution of the hydrophobic monomers on the backbone of the polymer. Inverse taper (IT) and blocky (B) sequences of hydrophobic moieties favor the formation of highly elastic interfaces after processing, while taper (T) and distributed (D) showed lower elasticity after processing, which is achieved by replacing good solvent with poor solvent and then nonsolvent. These structures allow for the study of the impact of the chemical composition and sequence of monomers on the properties of polymer-coated interfaces.

## Introduction

The amphiphilic nature of surfactants controls adsorption at fluid/fluid interfaces. Most surface-active molecules are composed of distinct parts that are compatible with the solvent (hydrophilic if the solvent is water) and incompatible with the solvent (hydrophobic if the solvent is water).^1,2^ If the chemical potential of surfactant molecules is higher in the bulk than at an interface, then surfactants are driven to adsorb.^3^ Adsorption leads to a loss of entropy due to the confinement of the molecules at a fluid–fluid interface. This loss of entropy is balanced by an enthalpic contribution that limits the number of contacts of the lyophilic parts of the surfactant molecules with the solvent.^4^ The chemical composition of surfactant molecules and the compatibility of each of its components with the solvent, therefore, are key factors determining adsorption. Surface-active polymers can be driven to adsorb to interfaces by decreasing the solvent quality. The solvent quality can be decreased further, which causes the polymers to collapse and form an insoluble, irreversibly adsorbed layer at the interface.^5,6^

Much of the existing literature on small-molecule surfactants describes their behavior at equilibrium. In an aqueous solution, surfactant self-assembly follows the rules of mass action.^7−10^ Surfactant molecules are distributed between the free solution and self-assembled structures according to a balance of reversible rates. The type of structure that forms is determined by a packing parameter, a geometric descriptor of the balance between hydrophilic head and hydrophobic tail.^11^ For example, an anionic surfactant, sodium dodecyl sulfate, transitions from spherical to ellipsoidal micelles with increasing ionic strength because of the reduction in size of the headgroup due to Debye screening from the background electrolyte.^12^ A tool to quantify the adsorption behavior of nonionic surfactants is the hydrophile–lipophile balance (HLB), originally developed to determine the type of oil/water emulsions formed using an ethylene-oxide-containing surfactant.^13^ HLB describes the mass fraction of the hydrophilic part of a nonionic surfactant on a scale of 0 to 20, with lower values being predominantly hydrophobic polymers suitable for antifoaming agents and water-in-oil emulsions and higher values being mostly hydrophilic polymers that work well as detergents, oil-in-water emulsifiers, and solubilizing agents.

As the molecular weight of surface active species increases and approaches polymeric surfactants, the available conformations of the molecules and intramolecular interactions need to be considered.^14−16^ As solvent conditions decrease from good solvent to nonsolvent during processing, the polymer molecules are trapped at the interface and undergo a chain collapse, limiting the contact of hydrophobic monomers with the nonsolvent. These collapsed structures have varying compressibilities depending on the conformation of the collapsed layer at the interface. Adsorption of polymers to interfaces and conformation at the interface are governed by the balance between the enthalpic interactions between surfactant and solvent molecules and the loss of entropy due to confinement to the interface. The nonionic copolymer surfactants consisting of nonadsorbing (favorable enthalpic interaction with solvent) and adsorbing (favorable enthalpy of adsorption) have been studied for many decades now and used in commercial products mostly as di- and triblock copolymers.^17^ Monomer sequences and interactions with solvents can be tailored and controlled by selecting side groups and adjusting the solvent quality.

At thermodynamic equilibrium, the adsorption of surfactant at an interface can be described by surface equations of state that relate the surface excess concentration, Γ, of surfactant at the interface to surface pressure, Π.^18,19^ Simple models assume that surface pressure linearly depends on the surface concentration, so as Γ increases, a commensurate increase in Π is observed. Surfactants have a maximum surface excess, Γ_∞_ concentration that is characteristic of molecular properties and needs to be determined with a direct measurement like neutron reflectometry.^20−22^ Methods such as neutron scattering are expensive and often difficult to access. Adsorption isotherms relate the amount of surfactant in the bulk, *C*_b_, to surface concentration, and are more easily obtained by measuring the decrease in interfacial tension as a function of bulk concentration. For an adsorbed surfactant to be at thermodynamic equilibrium, it must be free to ad/desorb completely.^23^ If the surfactant is reversibly adsorbed, then solution exchange causes interfacial tension to increase to its “clean” value (prior to any adsorption). For macromolecules, the more common situation is one in which surfactants adsorb irreversibly; that is, interfacial tension increases by a little or not at all during the removal of the bulk solution. In addition to the surface pressure, interfaces can be characterized by interfacial rheological properties, which depend on the adsorbed layer and its compressibility. By measuring these quantities, useful information about the amount of surfactant adsorbed to fluid/fluid interfaces can be gained without direct measurements, such as neutron reflectometry, while still quantifying the properties of the interfaces.

This paper directly probes the impact of the monomer sequence on the adsorption of irreversibly adsorbed surfactants at fluid/fluid interfaces. The polypeptoid molecules are stranded at the air/water interfaces by initial adsorption of the surfactant, followed by exchange of the surfactant solution with a nonsolvent. Four surface-active sequences of an amphiphilic polypeptoid are studied. Polypeptoid molecules are achiral, polypeptide analogs with substantially reduced hydrogen bonding. A highly precise control of the monomer sequence of copolymers offered by the advances in solid-state polypeptoid synthesis allows for the study of the conformation changes of macromolecule chains with different sequences in solution.^24−29^ Precise sequence specificity enables the construction of structure–property relationships at the interface for subtle changes in the order of hydrophilic and hydrophobic moieties. Here, we utilize this precise control of the molecular sequence in combination with interfacial characterization to investigate the relationship between the monomer sequence and qualitative differences in adsorbed layers.

## Materials and Methods

**Figure 1:**
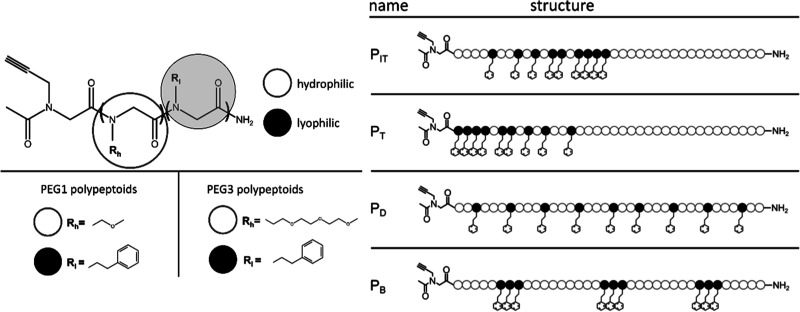
Chemical structure (left) of polypeptoid molecules for the four sequences studied. All four sequences have the same ratio of hydrophilic to lyophilic groups (27:9) with a molecular weight of 4712 g/mol (PEG1, with the hydrophilic side group being one repeat unit of polyethylene glycol) and 7092 g/mol (PEG3, more hydrophilic, with three repeat units of polyethylene glycol as a hydrophilic side group), respectively. The naming conventions of the sequences (right) are described in terms of the distribution of the lyophilic (hydrophobic) side groups starting from the propargyl end group: inverse taper (P_IT_), taper (P_T_), distributed (P_D_), and blocky (P_B_).

Figure 1 shows the structure of the four polypeptoid sequences used in this paper: inverse tapered (P_IT_), tapered (P_T_), distributed (P_D_), and blocky (P_B_), and these definitions have been used in other studies.^27,30^ The naming convention is based on the distribution of hydrophobic (aromatic-containing) moieties in the backbone.

Polypeptoids were synthesized leveraging an automated Prelude peptide synthesizer using standard methods and commercially available reagents.^30,31^ Specifically, PEG1 polypeptoids were prepared as described by Segalman and co-workers.^27^ The PEG3 sub-monomer (2-(2-(2-methoxyethoxy)ethoxy)ethanamine) is bulkier than the PEG1 sub-monomer (2 methoxyethylamine); the amine displacement steps were extended from 1 to 2 h during the synthesis of PEG3 polypeptoids. All other synthetic steps were performed, as previously described.^27^ The resulting PEG3 polypeptoids were purified by preparative high-performance liquid chromatography, and the identity of the desired product was confirmed by liquid chromatography–mass spectrometry; results for the PEG3 set are shown in Table 1.

**Table 1:** Formation of the Desired Polypeptoid Product Is Confirmed by Observation of the m/2 + 2H^+^ Fragment^a^

sequence	calc (g/mol)	observed (*m*/*z*)
PEG3-P_IT_	7092.31	3547.2
PEG3-P_T_	7092.31	3547.2
PEG3-P_D_	7092.31	3547.1
PEG3-P_B_	7092.31	3547.1

a Results for the PEG3 monomer.

Both PEG1 and PEG3 polypeptoids readily dissolve in pure acetonitrile but are insoluble in water. To control solvent conditions, samples are prepared in good solvent conditions (φ_ACN_ = 0.5), and then the content of water is increased to reduce solvent quality. The values of clean surface tension for different volume fraction mixtures of acetonitrile and water without the presence of surfactant, crucial for calculating surface pressure, were measured and are included in Figure S1 in the Supporting Information; the surface tension of water and ACN against air are 72.2 and 29.3 mN/m, respectively.^32^

Acetonitrile (ACN, 99%+) was purchased from Sigma. Deionized water, termed water, with a resistivity of 18.2 MΩ·cm was produced using a Barnstead Ultrapure water filtration system. Mixtures of water and acetonitrile are prepared on a volumetric basis. For example, a φ_ACN_ = 0.25 is 25% ACN and 75% water by volume, assuming ideal volumetric mixing. The φ_ACN_ = 0.25 mixture contains just enough ACN to solubilize the polypeptoids. Lyophilized polypeptoids were weighed, and solvent (φ_ACN_ = 0.5) was added to dissolve the material and make 0.4 mM stock solutions. Samples diluted by a factor of 4 (for the final *c* = 0.1 mM) were used during experiments. Although a cmc for these polypeptoids was not measured, at this concentration, the radius of gyration was consistent for single-chain polypeptoids with a degree of polymerization of 36.^27^ All solutions were prepared in acid-washed vials.

**Figure 2:**
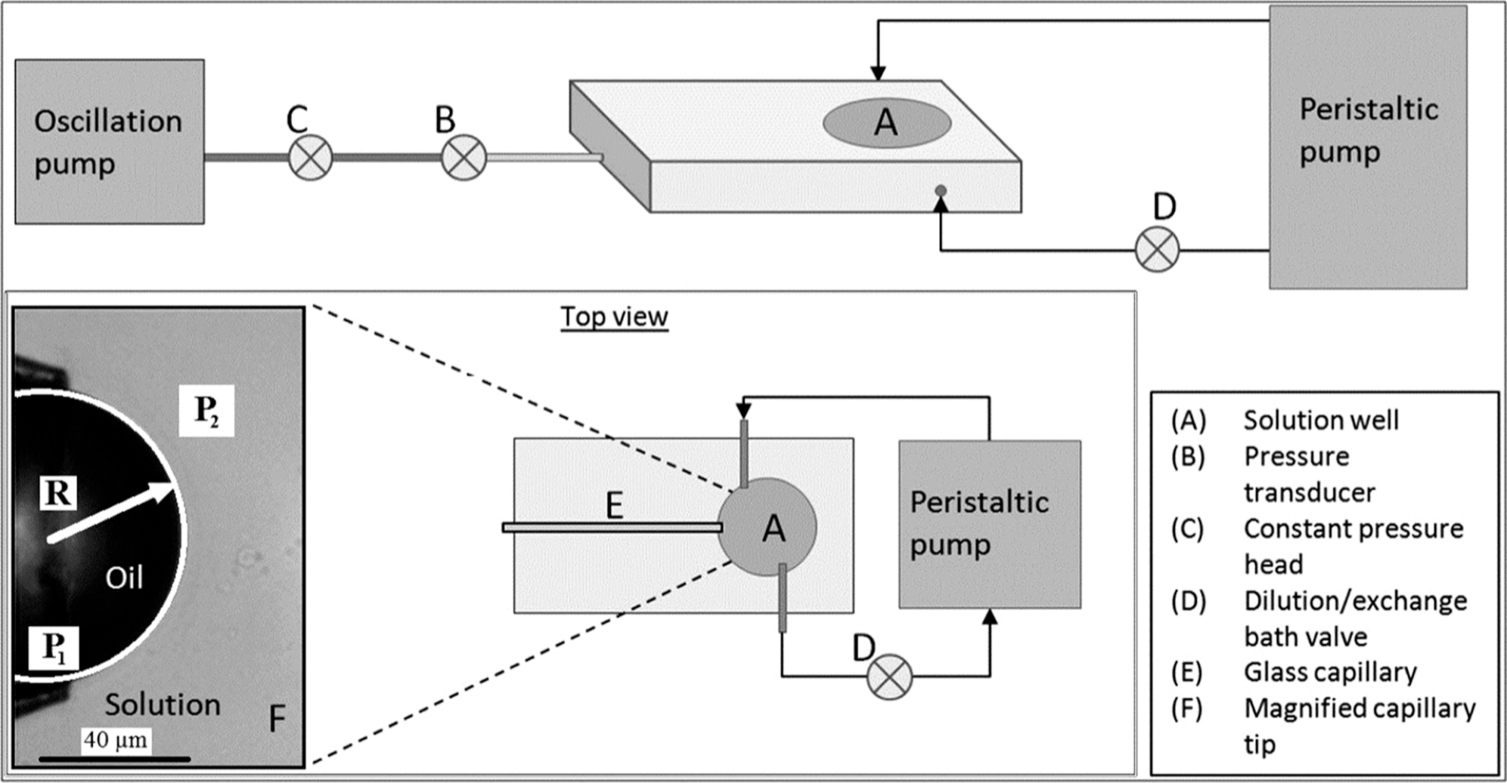
Schematic representation of the microtensiometer adapted from ref (33). A solution well (A) contains the polypeptoid solution. Pressure transducer (B) is used to measure the back pressure, *P*_1_, inside the capillary I and hydrostatic pressure of the constant pressure head (C), and *P*_2_ is determined based on the volume and density of the fluid above the tip of the capillary (F). A peristaltic pump (D) is used to exchange the contents of the solution well to process the interfaces.

Interfacial properties are measured with a microtensiometer platform, described in detail elsewhere.^34−39^ Based on a capillary tensiometer, separate measurements of both the pressure jump across the interface and the curvature of the interface allow for rapid characterization of interfacial tension without the need for shape fitting or reliance on distension by gravity. The use of microscale interfaces also has the advantage of requiring smaller amounts of material and the ability to alter the bulk solution rapidly in comparison to surfactant dynamics. The schematic is provided in Figure 2. A polypeptoid solution is poured into the 3 mL solution reservoir. A glass capillary of a radius between 45 and 30 μm is inserted into the reservoir, and it is connected to a pressure transducer. The air/water interfaces are pinned at the tip of the capillary and are imaged on a Nikon T-300 inverted light microscope. The radius of curvature is determined by using a LABVIEW routine. The interfacial tension is calculated with the Laplace relation given in eq 1
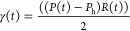
1where *P*(*t*) is the Laplace pressure difference across the interface as a function of time, *P*_h_ is the hydrostatic pressure, *R*(*t*) is the radius of the interface as a function of time, and γ(*t*) is the calculated dynamic interfacial tension. All interfaces in this current work are between an air-filled, hydrophobized capillary and a reservoir containing aqueous solutions (water or a mixture of acetonitrile and water). The microtensiometer platform offers a few advantages over other methods for studying fluid interfaces. The solution reservoir requires a small volume of a sample (1 to 5–4 mL) requiring only micrograms of polypeptoids for each experiment. Additionally, the small surface area (about 2000 μm^2^) and high curvature of the interfaces drive fast adsorption. This allows an experiment with multiple steps to achieve a new steady state to require a few dozen minutes instead of multiple hours.

The microtensiometer reservoir has two lines connected to a deionized water reservoir and waste, respectively, that are controlled with a peristaltic pump. The processing of interfaces is conducted by exchanging the solution of polypeptoid dissolved in a mixture of water and acetonitrile with pure deionized water at a rate of a full reservoir volume exchange every 20 s. Water and ACN have drastically different values of equilibrium surface tension with air (see Figure S1). The equilibrium surface tension values of the three mixtures utilized are much closer to the surface tension of pure acetonitrile than that of water. In the absence of surface-active components, the equilibrium surface tension of the ACN/water mixtures develops instantaneously and does not change with time.

Interfacial dilatational rheology is measured by inducing small-amplitude oscillatory dilatations (Δ*A* ≈ 3%), as well as large-amplitude (Δ*A* ≈ 10%) expansion–compression cycles. Dilatational modulus is defined in eq 2 as the change in surface excess normal stress *P*^S^ with interfacial area *A*
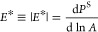
2

The area of the hemispherical caps is calculated using eq 3 as
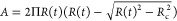
3where *R* is the radius of curvature of the interface, and *R*_c_ is the radius of the glass capillary. The large amplitude compression modulus (*E*_c_) is determined from the slope of the best linear fit of interfacial tension versus the normalized surface area of the interface given by using eq 4
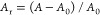
4

## Results

**Figure 3:**
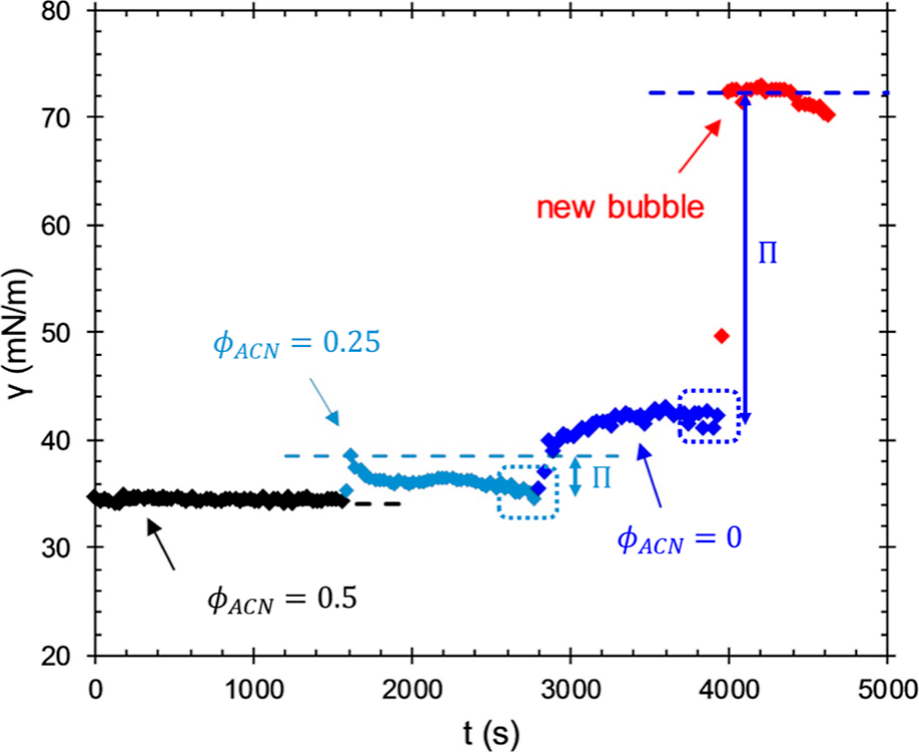
Dynamic surface tension of an air/liquid interface during exposure to 0.1 mM P_IT_ PEG1 in φ_ACN_ = 0.5 [◆ (black)], after dilution of the P_IT_ solution to 0.05 mM in φ_ACN_ = 0.25 [◆ (light blue)], during continuous exchange of the P_IT_ solution with water [◆ (blue)], and of a fresh interface formed in the exchanged reservoir [◆ (red)]. The dashed horizontal lines denote the clean surface tension of the solvent mixtures without P_IT_. The dotted rectangles indicate the time of measurement of the dilatational modulus of each interface.

We conducted a series of experiments on eight polypeptoid surfactants (2 compositions, 4 sequences) to determine the properties of interfaces. Figure 3 shows the experimental procedure followed for all of the samples. Figure 3 demonstrates our procedure for the preparation and characterization of adsorbed layers of an air/solution interface in a reservoir of an aqueous solution of 0.1 mM P_IT_ of PEG1 in φ_ACN_ = 0.5. At time *t* = 0 s, a fresh interface is created and equilibrated (black diamonds). The interfacial tension is calculated using eq 1 at a rate of 30 fps. The interface instantaneously develops a surface tension of ∼35 mN/m that remains unchanged for 1600 s. The dashed, black line denotes the equilibrium surface tension of the φ_ACN_ = 0.5 solvent mixture against air (without polypeptoid); the surface tension of the ACN/water mixtures against air is provided in Figure S1. The overlap between the data points and dashed line for a full 1600 s shows that 0.1 mM P_IT_ does not change the surface tension, so the polypeptoid molecules likely do not adsorb to the air/solution interface from a φ_ACN_ = 0.5 mixture. This is consistent with the high solubility of these polypeptoid molecules in ACN.

At *t* = 1600 s, 1 mL of water is added to the reservoir that contains 1 mL of P_IT_ solution and mixed. The addition of water decreases the concentration of P_IT_ from 0.1 to 0.05 mM and changes the solvent mixture from φ_ACN_ = 0.5 to φ_ACN_ = 0.25. This increases the surface tension of the air/solution interface initially to ∼39 mN/m, followed by a decrease to 35 mN/m over 1000 s (light blue diamonds). The dashed light blue line denotes the increase in equilibrium surface tension that accompanies a change in φ_ACN_ (see Figure S1 in Supporting Information). A decrease in surface tension to a value below the clean solvent value shows that the P_IT_ is surface active in the φ_ACN_ = 0.25 mixture and that some amount of polypeptoid molecules adsorb to the interface.

At t = 2800 s, the 0.05 mM P_IT_ solution is exchanged continuously with water (τ_R_ = 10 s, *V* = 3 mL) until *t* = 3800 s, where flow is ceased for an additional 200 s (dark blue diamonds). At the beginning of the rinse, the reservoir solution becomes turbid with visible, microscopic aggregates of bulk PEG1 polypeptoid molecules before rapidly clarifying. No turbidity was observed during water rinse for the more water-soluble PEG3 polypeptoid molecules. Surface tension increases sharply from the prerinse value of 35 mN/m (*t* = 2800 s) and eventually reaches a plateau near γ = 42 mN/m. At this point, ACN has been removed from the reservoir, and the equilibrium surface tension of the reservoir solution (water) is 72 mN/m, given by the dashed blue line. The after-rinse plateau value of γ = 42 mN/m is significantly lower than the surface tension of pure water (γ = 72 mN/m), demonstrating that P_IT_ formed an irreversibly adsorbed layer on the interface. At *t* = 4000 s, the existing interface is ejected to create a fresh, test interface (red diamonds). This is done to determine if any P_IT_ remains in the reservoir after rinsing. The surface tension of the test interface coincides with the clean value of pure water for approximately 400 s before beginning to drift downward, signifying some adsorption, likely from polypeptoids remaining and desorbing from the cell walls. After being aged for 600 s, the test interface reaches a surface tension near 70 mN/m. While the rinse did not remove all surface-active material from the reservoir surfaces, the slow adsorption and modest surface tension (γ = 70 mN/m) of the test interface greatly contrast with the substantially lower after-rinse value (γ = 42 mN/m), demonstrating that the plateau in surface tension during the water rinse results from irreversibly adsorbed P_IT_ rather than a new equilibrium with a diluted bulk solution.

Figure 3 shows that P_IT_ can be used to create processable interfaces. Adsorption is driven by solvent quality, a 0.1 mM solution in a better solvent (φ_ACN_ = 0.5) shows no adsorption, whereas a more dilute, 0.05 mM solution in a poorer solvent (φ_ACN_ = 0.25) does show adsorption. When the P_IT_ solution is exchanged with a nonsolvent, water, the value of the surface tension reaches a plateau at 42 mN/m, a surface pressure value of about 30 mN/m. While it is certain from the results of the test interface that this plateau signifies irreversibly adsorbed P_IT_, it is unclear whether the interface after rinsing is exclusively comprised of P_IT_ present before rinsing or whether the precipitous decline in solvent quality drives additional adsorption beyond what is seen from the φ_ACN_ = 0.25 mixture. Given that P_IT_ adsorbs only modestly from the φ_ACN_ = 0.25 mixture but remains strongly adsorbed after the rinse, the latter explanation is more likely.

All subsequent experiments are carried out with a procedure similar to the one shown in Figure 3. An air/solution interface is first equilibrated against a 0.1 mM polypeptoid solution in an φ_ACN_ = 0.25 mixture (poor solvent) for 1000 s. The equilibrated interface is then rinsed with water (τ_R_ = 10 s, *V* = 3 mL) for 1000 s and allowed to rest without flow for 200 s. The interface is then jettisoned to produce a fresh interface, which is used to test the composition of the reservoir after the rinse. Both before and after rinsing, dilatational, and compression moduli are measured (see the dotted rectangles in Figure 3).

**Figure 4:**
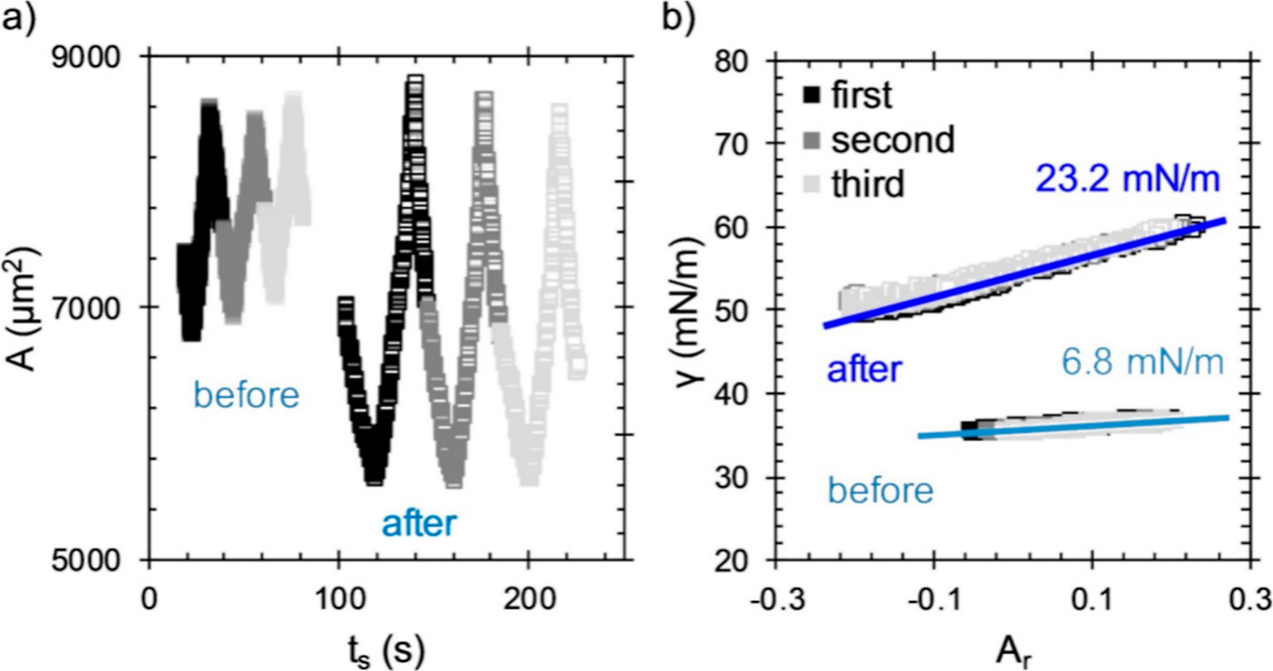
Surface area (a) and surface tension (b) during large amplitude compression before (filled points) and after rinsing with water (empty points) of the PEG1 P_T_-laden interfaces. Values of *E*_c_ calculated from these data are given in (b). The oscillations were performed at a rate of roughly 50 μm^2^/s.

Figure 4 shows the large amplitude compressions of an interface with adsorbed P_IT_ before and after a rinse. Figure 4a shows the interfacial area during the compression cycles. The points have been shifted along the horizontal axis to conveniently separate data before (filled points) and after rinse (empty points), now plotted against the shifted time, *t*_s_, on the same axes. Interfacial compression and expansion occur at a rate of 50 μm^2^/s (using a syringe pump). Each interface is compressed and expanded three times with the first, second, and third compressions given by the black, dark gray, and light gray points, respectively.

A larger magnitude of Δ*A* can be applied to an interface after the rinse than before the rinse. The pretreatment of the capillary (hydrophobization of the glass interface) required to achieve suitable pinning of an air/water interface performs less well with ACN/water mixtures and larger amplitudes than those shown to cause the air/solution/glass contact line to shift during compression, invalidating the measurement.

Figure 4b shows the variation of surface tension (determined using eq 1) during compression with the nondimensionalized interfacial area using eq 4, calculated by normalizing the area by its value prior to compression, *A*_0_. Both before and after rinse, the first, second, and third cycles show no hysteresis. Hysteresis is not observed for any of the compression cycles in this study, demonstrating that these interfaces are capable of recovering reversibly from large strains. The magnitude of the compression modulus is extracted from the slopes of the data and is calculated by linear regression. The best fit lines and values for the effective compression modulus, *E*_c_, are shown in Figure 4b. Before rinsing, the value of *E*_c_ is low (6.8 mN/m) and increases to a value of 23.2 mN/m after replacing the bulk phase with water. The compression moduli of all the polypeptoid-laden interfaces studied here increase after rinsing adsorbed polymer layers, suggesting that these layers have become less compressible with processing. We use the slope as a measure of the compression moduli, which yields the same result as a formal fit to a constitutive equation.^40^

**Figure 5:**
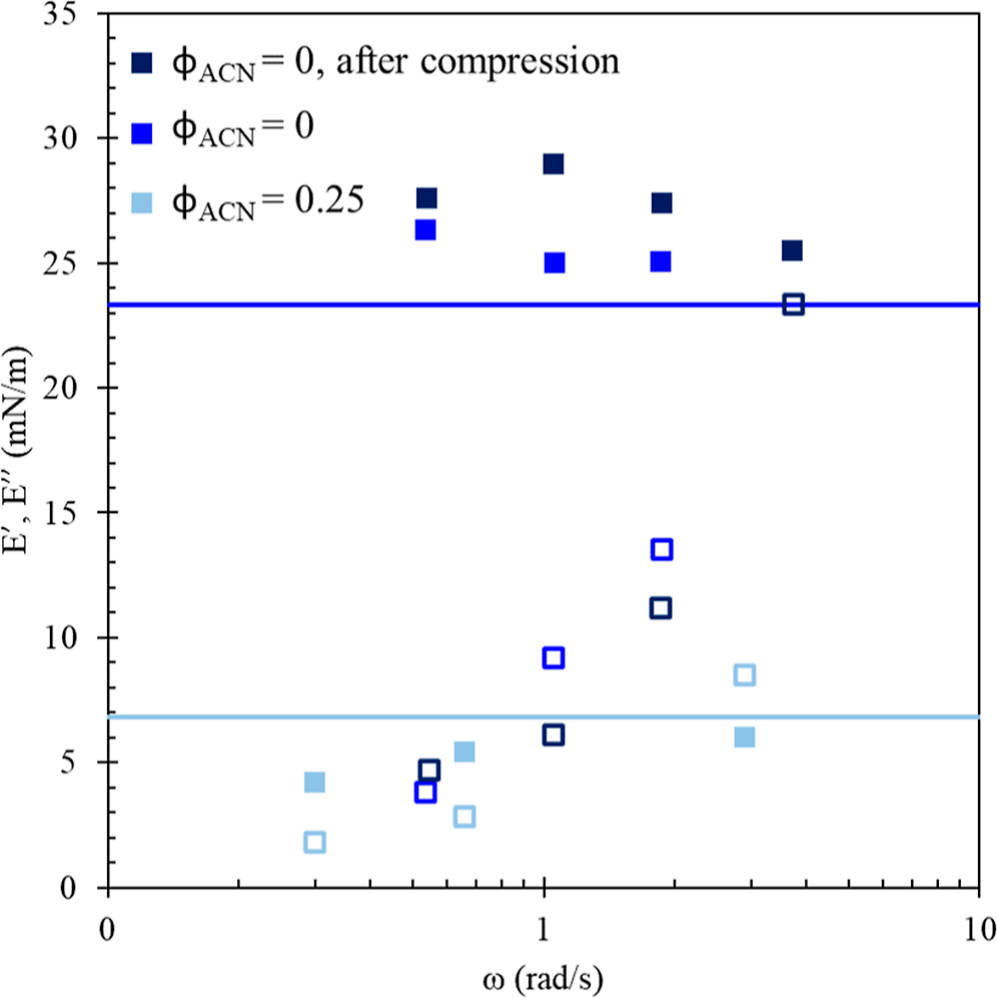
Elastic [■ (black)] and viscous [□ (black)] components of dilatational modulus after adsorption from 0.1 mM PEG1 P_T_ in φ_ACN_ = 0.25 [■, □ (light blue)], after rinsing with water [■, □ (blue)] and following the after-rinse large-amplitude compressions [■, □ (dark blue)]. The light blue and dark blue lines give values of compressional modulus before and after rinsing, respectively, provided in Figure 6 (square symbols).

Figure 5 shows the real (filled symbols) and imaginary (empty symbols) components of the dilatational modulus measured under small amplitude oscillatory dilatation for the interfaces characterized in Figure 4. Before the rinse (light blue symbols), both elastic and viscous moduli are small (<10 mN/m) over the range of frequencies used, 0.3 < ω < 3 rad/s. Both moduli show a negligible dependence on frequency, and both have values near the compression modulus (light blue horizontal line), *E*_c_ = 6.8 mN/m. After rinsing (blue symbols), the elastic modulus is substantially larger than before the rinsing and depends only weakly on frequency, *E*′ = 28 ± 2 mN/m. The value of the viscous modulus after rinse increases with increasing frequency, at low frequencies near the prerinse values *E*″ (ω = 0.55 rad/s) = 5 mN/m and at high frequencies near that of the elastic modulus *E*″ (ω = 4 rad/s) = 27 mN/m. After rinse, the value of the elastic modulus is close to the compression modulus, and the viscous modulus is less than *E*_c_ at all but the largest frequency, ω = 4 rad/s.

The dilatational modulus is measured in the small amplitude, or linear, limit prior to the large compressions shown in Figure 4. To determine if the measurement of the compression modulus is itself processing the interface and changing its properties, the dilatational modulus has been measured again after rinse and after the large amplitude compressions. The dark blue points in Figure 5 show good quantitative agreement with the after-rinse data prior to compressions (blue points), showing that the effects of the large amplitude (and likely nonlinear) compressions used to measure *E*_c_ do not linger beyond the measurement. This is consistent with the lack of hysteresis observed in Figure 4 and with the small values of the viscous modulus at low frequencies in Figure 5. Viscous contributions at low frequencies are quite small, resulting in an in-phase stress response at all but the highest frequencies probed. At the highest frequency measured, we see deviations that are attributed to syncing issues between the two signals, so we focus on results at ω = 1 rad/s, where we have the most confidence in the decoupling of the in-phase and out-of-phase signals.

**Figure 6:**
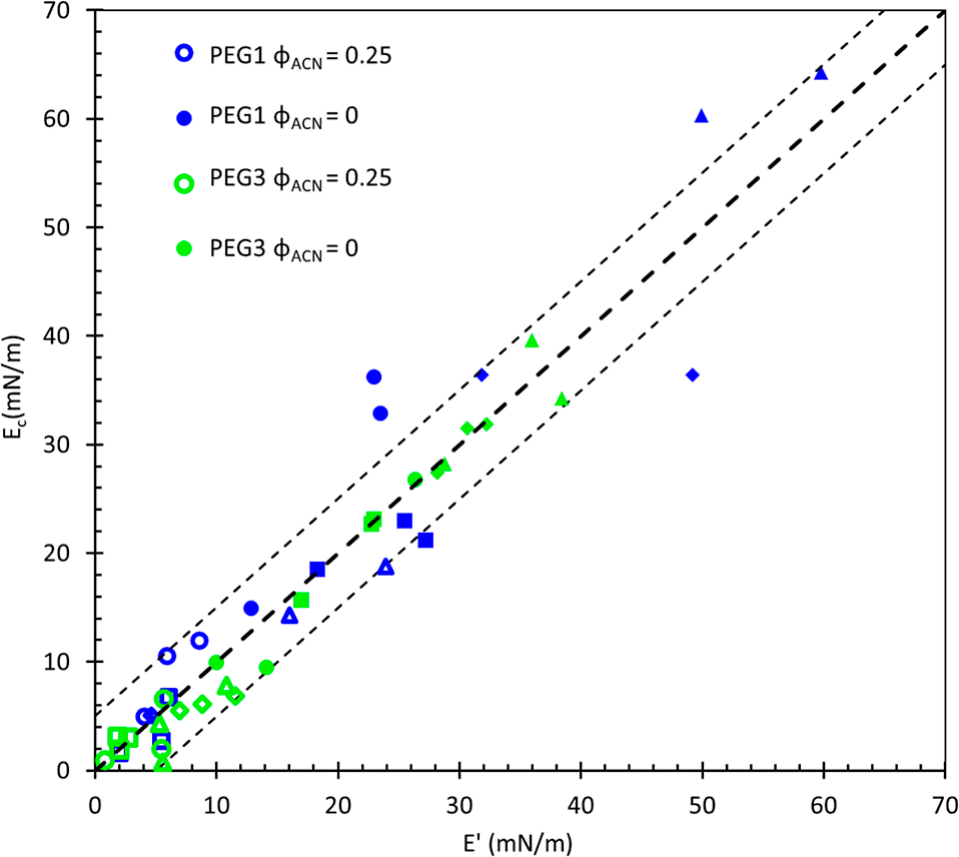
Comparison of compression modulus and elastic modulus at a frequency of 1 rad/s after adsorption from a 0.1 mM solution in φ_ACN_ = 0.25 (empty symbols) and after water rinse (filled symbols) for PEG1 (blue) and PEG3 (green) polypeptoids. Different shaped symbols represent different sequences of polypeptoids: blocky [▲ (blue, green)], distributed [● (blue, green)], inverse taper [◆ (blue, green)], and taper [■ (blue, green)]. The thick dashed line is a guide for the eye, representing perfect agreement, and the thinner dashed lines show a ±5 mN/m interval.

A comparison between the elastic and compression moduli is provided in Figure 6. Agreement between *E*_c_ and *E*′ across all frequencies suggests that dilatational stresses are not relaxed by material exchanging with the interface during oscillation. Many small-molecule surfactants adsorb reversibly and are capable of exchange with the adjacent solution during interfacial compression/expansion, often at a rate limited by diffusion.^41−43^ Exchange acts both to lower the measured value of the dilatational modulus and to push the stress response to one dominated by the out-of-phase, viscous component. Instead, the elastic modulus is constant across all frequencies, dominates at low frequencies, and coincides with the compression modulus (the low frequency limit), contrasting with an explanation of relaxation by a diffusional exchange, which would suppress the elastic response at lower frequencies. It is not surprising that the after-rinse moduli do not exhibit material exchange, given that water is a nonsolvent for these polypeptoids. The data show that the viscous modulus contributes more significantly at higher frequencies, a response that is not predicted by models of diffusional exchange. These dilatational data are better described by a constitutive model focused on contribution from interfacial rheology.^44,45^ This figure compiles data from all of the polypeptoids studied here to allow for comparison of different moduli measurements. A discussion of the differences in properties of these polypeptoid systems follows.

**Figure 7:**
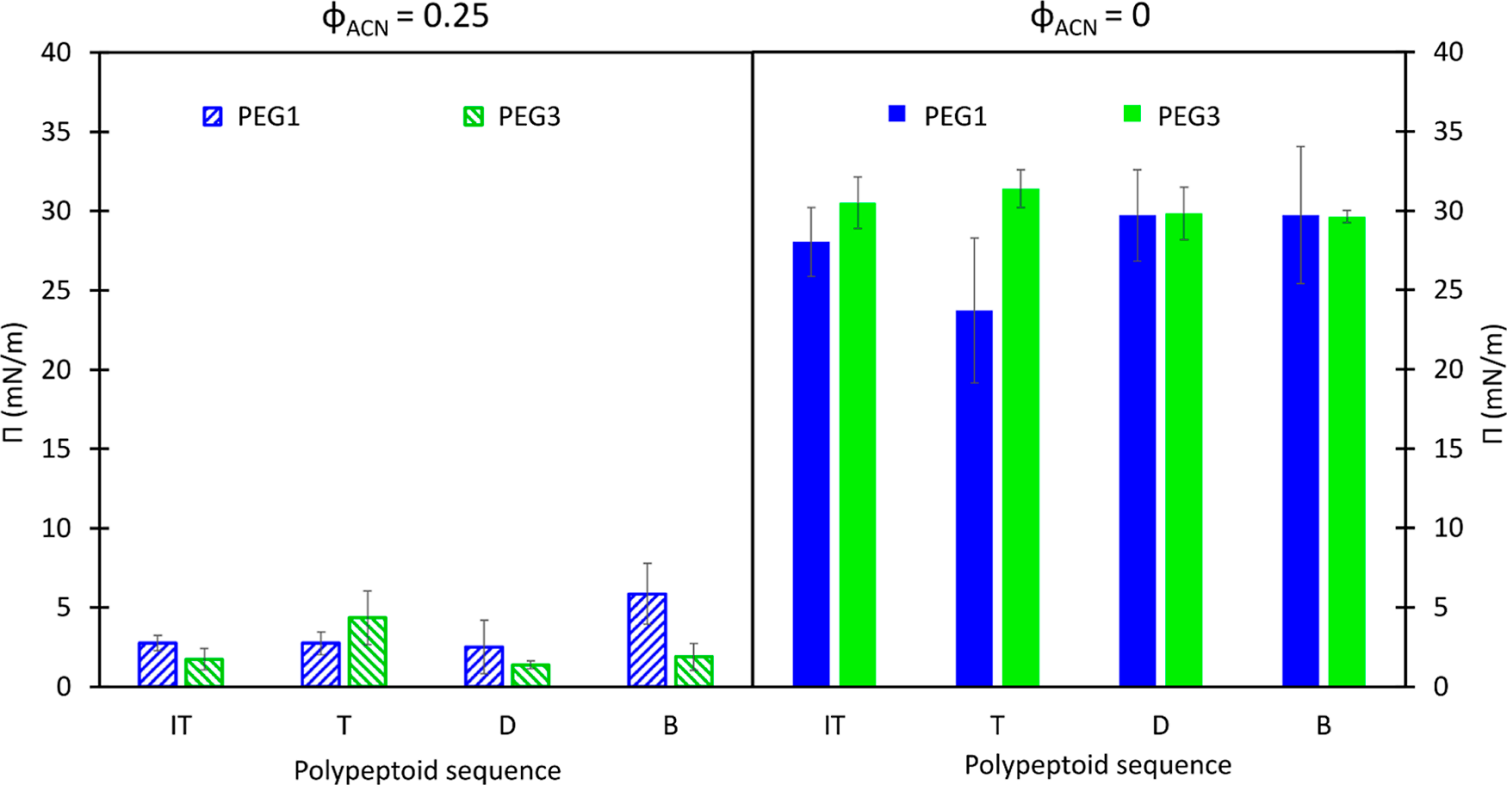
Surface pressure before (φ_ACN_ = 0.25, diagonal stripes) and after (φ_ACN_ = 0, solid fill) rinsing with deionized water of polypeptoid-laden air/water interfaces with PEG1 (blue) and PEG3 (green). Error bars are standard deviations from three measurements.

Figure 7 shows surface pressure for all four sequences before (a) and after rinse (b), with error bars providing standard deviations across at least three replicates. Values of surface pressure measured against differing solvents (before and after rinse) do not show significant differences among sequences. Each polypeptoid adsorbs modestly from an initial 0.01 mM solution at φ_ACN_ = 0.25. Exchanging the polypeptoid solution with water causes surface pressure to increase by ≈25 mN/m to a final value of about 30 mN/m for all sequences. There is not a significant difference between PEG1 and PEG3 surface activity after the initial adsorption at φ_ACN_ = 0.25 or after the rinse (φ_ACN_ = 0), despite PEG3 having a higher molecular weight and being slightly more water-soluble. As stated before, the surface pressure depends on the amount of surface-active material adsorbed at the interface. The measured values of surface pressure are different before and after rinse but very similar to each other for the samples of the same chemical composition but different monomer sequences. The polymers are composed of hydrophilic groups (soluble in the solvent) and hydrophobic groups (insoluble), which drive adsorption to the interfaces. Therefore, these results indicate that the chemical composition of the hydrophilic moieties is not the main factor determining the surface activity of these molecules. Rather, the irreversible adsorption of the hydrophobic side groups seems to be driving the adsorption of this composition of polypeptoid molecules. Notably, the sequence of the hydrophobic moieties on the backbone of the molecules does not affect the surface concentration of adsorbed layers. For this interpretation, we assume that the surface pressure is proportional to the surface concentration; this is an assumption in this work, but we have provided a motivation and path to design a neutron reflectivity or other quantitative measurement to verify. Without this work, it would be difficult to justify that measurement or design the experiment for success.

**Figure 8:**
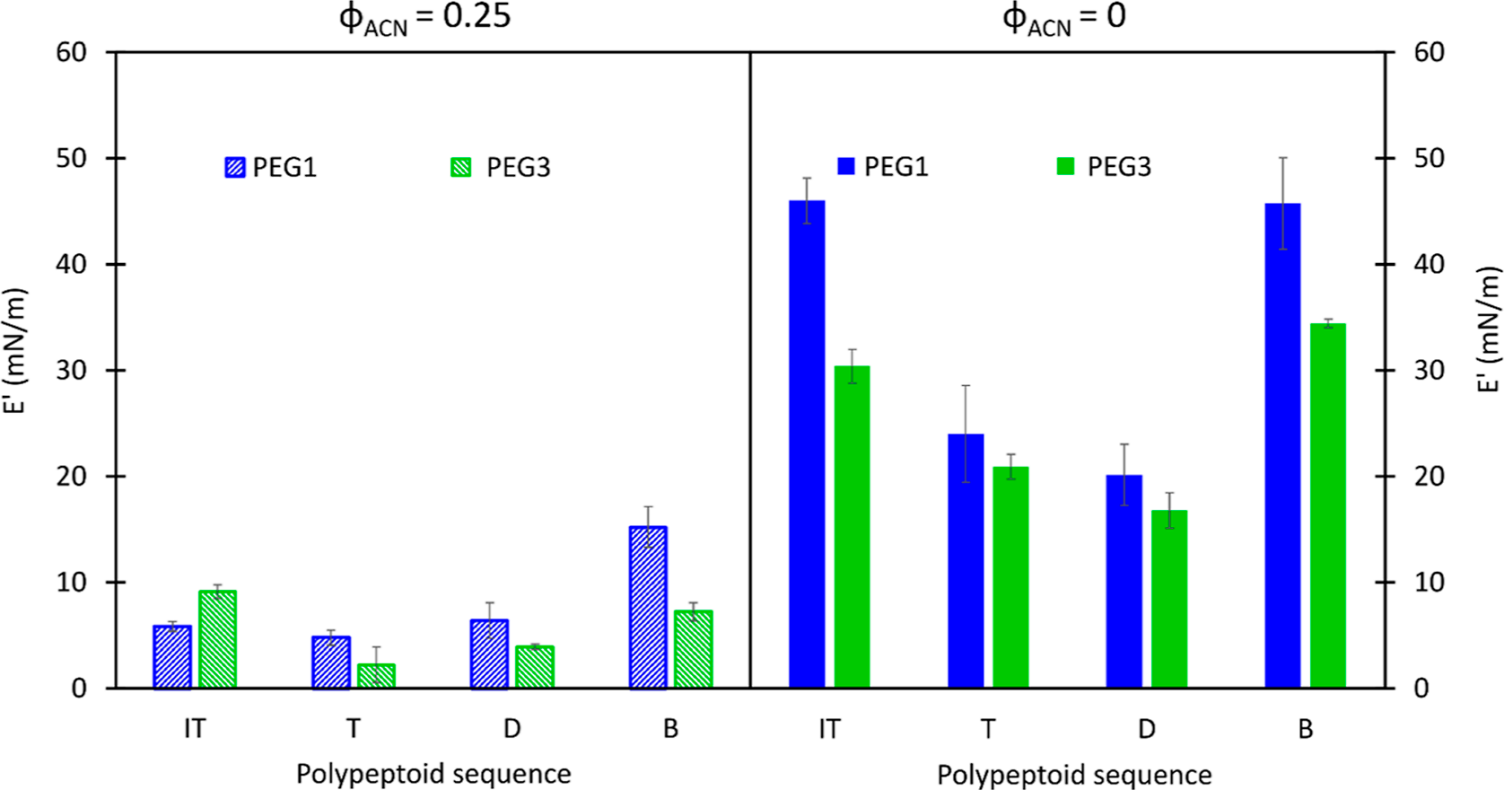
Elastic modulus *E*′ before (a, φ_ACN_ = 0.25) and after rinse (b, φ_ACN_ = 0) with deionized water of polypeptoid-laden air/water interfaces with PEG1 (blue) and PEG3 (green). Error bars are standard deviations from three measurements.

While the surface pressure does not show sequence dependence, the sequence does affect dilatational moduli, which we equate to the compressibility of the layers. Figure 8 shows the elastic modulus at 1 rad/s for polypeptoid-laden interfaces (a) before and (b) after rinsing for PEG1 and PEG3 polypeptoid molecules. Before rinsing, the elastic modulus of all sequences is small, *E*′ < 20 mN/m. Elastic modulus increases with rinsing, with values up to 60 mN/m for PEG1 and up to 35 mN/m for PEG3 polypeptoid molecules. The after-rinse values of elastic modulus show a strong sequence dependence. Both the inverse taper and blocky sequences have higher after-rinse moduli than the tapered and distributed sequences, with the inverse tapered and distributed moduli having a statistically significant difference (*p* < 0.05).

This relationship between postprocessing dilatational elasticity is preserved between the two chemistries studied, as we see that the elastic modulus, *E*′, of P_B_ and P_IT_-laden interfaces are consistently higher than P_D_ and P_T_ for both PEG1 and PEG3. The increase in elastic modulus with rinsing is not correlated with the increase in surface tension beyond the general, positive trend. Figure 6 demonstrates that we will observe the same trends using the large amplitude compressive moduli.

## Discussion

A surface equation of state relates the amount of adsorbed material to an interface (Γ, surface excess concentration) and the energy of the interface (γ, surface tension).^2^ Typically, surface tension is measured over a range of solution concentrations, combined with direct measurements of surface concentration for a single surfactant, and used to determine both an equation of state and an adsorption isotherm, relating surface excess and solution concentration. Given the similarity in before-rinse values of surface tension shown in Figure 7 all at the same solution concentration, it is not unreasonable to assume that these four sequences have similar surface excess concentrations in the φ_ACN_ = 0.25 mixture. Exchanging the solvent mixture with water increases surface tension similarly for the four sequences, suggesting that the surface concentration does not change and similar changes in the surfactant structure can be expected. The water rinse is done at a rate corresponding to a full reservoir exchange every 20 s, which is comparable to a time scale of initial adsorption to the interfaces at *c* = 0.1 mM, so it cannot be ruled out that additional adsorption to the interfaces occurs during this process. However, the rate of adsorption depends on the bulk concentration, which decreases during the rinse, which counters that effect. Therefore, assuming that before-rinse adsorption and additional adsorption during the rinse are comparable for all sequences requires that the after-rinse surface excess concentration also be comparable. These two sets of four polypeptoid molecules have the same chemical composition (PEG1 and PEG3 based on the hydrophilic side groups, respectively) and showed similar surface activities, despite the differences in monomer sequences between different sequences. The solvent exchange likely induces a change in the conformation of the polypeptoid molecules at the interface. Based on the surface pressure data, the amount of each monomer sequence in the polypeptoid molecules is the same for the same chemical composition; the differences in mechanical properties reflect the interactions of the polymer molecules with each other due to different conformations after processing.

After-rinse dilatational elasticity varies with the sequence, with P_IT_ and P_B_ having the largest values. Assuming that surface concentrations are comparable, the difference in dilatational stress response is best explained by specific intermolecular interactions rather than by the number of interacting molecules (if surface concentrations vary). The intermolecular interactions between adsorbed molecules will depend on sequence and orientation. P_B_ has a larger after-rinse modulus than P_D_, although both have hydrophobic groups somewhat distributed along the chain. Because the hydrophobic groups of P_B_ are grouped in sequences of three, P_B_ is likely to adopt a conformation at the interface flatter that is than that of P_D_. In other words, thermal energy is more likely to pull a single phenyl ring into the water than three. As a result of the flatter conformation, a single P_B_ molecule would occupy more space, increasing the likelihood of interaction between neighboring chains via hydrophobic interactions (e.g., π–π stacking).^46^

P_IT_ should adopt a conformation that is flatter than that of P_T_ for similar reasons. The hydrophobic groups of P_T_ are tapered in a way that resembles a traditional one-tailed surfactant copolymer, whereas the hydrophobic groups of P_IT_ are biased toward the center of the chain in a way that resembles an asymmetric, ABA triblock copolymer (where block A is soluble and block B is selective). An ABA triblock copolymer occupies more space at an interface than a diblock copolymer due to a phenomenon known as dangling tails.^47,48^ Essentially, a triblock copolymer of the same mass as a diblock packs less efficiently because the diblock can more easily form a brush in the solvent, lowering the size of its adsorption site. For the same number of adsorbed molecules, P_IT_ would lie flatter than P_T_ and have stronger intermolecular interactions because of it. Based on the similar values of surface pressure, we can say that there are similar amounts of molecules of each sequence at the interface. Therefore, a flatter conformation would leave less space between the molecules at the interface and cause them to interact more as the interface is compressed/expanded, which is reflected in higher values of dilatational modulus for P_B_ and P_IT_ than P_D_ and P_T_, respectively.

The elasticity of PEG3-laden interfaces is consistently lower than that of those with less hydrophilic PEG1. The effect of the sequence is preserved, indicating that the monomer sequence is affecting the conformation of the molecules within the processed layer. The values of the dilatational modulus, however, are consistently lower. Again, with a similar amount of molecules per area of interface, PEG3 would need to have greater spacing between molecules due to a less flat conformation, or, in other words, extending further into the bulk solution and away from the interface. This is consistent with the differences in chemical composition between PEG1 and PEG3, with the hydrophilic moieties of PEG3 being more hydrophilic and, therefore, being enthalpically favored to interact with the outer phase (water) more.

## Conclusions

Solvent quality controls the adsorption of amphiphilic polypeptoids. The four sequences and two different chemical composition of polypeptoids studied here are not surface active in a good solvent, a 50/50 mixture of ACN/water. These polypeptoids adsorb modestly (lower γ by ≈5 mN/m) to the air/liquid interface from a 0.1 mM solution in a poor solvent, 25/75 ACN/water. After adsorption, the exchange of the polypeptoid solution with pure water strands the molecules at the interface; surface tension increases slightly but remains ∼30 mN/m below the clean value of air/water, 72 mN/m. Differences in monomer sequence and increasing the size of hydrophilic groups do not have a first-order effect on surface activity. The small dilatational elasticity before the water rinse (*E*′ < 20 mN/m) increases by at least a factor of 2 with the exchange, up to 60 mN/m. Agreement between the values of elastic and compression moduli suggests that material exchange between the interface and the solution is not responsible for relaxing dilatational stresses. The values of the after-rinse elastic modulus show a dependence on the molecular sequence, with P_IT_ and P_B_ forming more elastic interfaces than P_T_ and P_D_. P_IT_ and P_B_ sequences likely adopt flatter conformations at the interface, enabling their hydrophobic groups to interact more strongly than P_T_ and P_D_. The trends are preserved for both PEG1 and PEG3 polypeptoids, indicating that the arrangement of the hydrophobic moieties has the strongest impact on the adsorption and elasticity of the interfaces. The hydrophilic part can be changed while still preserving surface activity. Since the conformation changes responsible for the increasing elasticity of the interfaces are due to the thermodynamic interactions between the polymer monomers and the solvent, a similar effect should be possible to achieve with factors other than solvent quality, such as temperature. This provides flexibility and guiding principles in designing processable polypeptoid surfactant molecules by tailoring the interactions with the solvent and the final properties of interfaces.

## Supporting Information Available

Surface tension of acetonitrile/water mixtures measured with the microtensiometer (PDF)

## References

[ref1] Hiemenz P. C. Principles of Colloid and Surface Chemistry, 2nd ed.; Marcel Dekker, Inc.: New York, 1986.

[ref2] Berg J. C. An Introduction to Interfaces and Colloids: The Bridge to Nanoscience; World Scientific Publishing Co. Pte. Ltd.: Danvers, 2010.

[ref3] Bergfreund J.; Siegenthaler S.; Lutz-Bueno V.; Bertsch P.; Fischer P. Surfactant Adsorption to Different Fluid Interfaces. Langmuir 2021, 37 (22), 6722–6727. 10.1021/acs.langmuir.1c00668. 34030438

[ref4] Lambourne R.; Strivens T. A. Paint and Surface Coatings: Theory and Practice, 2nd ed.; Elsevier Science, 1999.

[ref5] Rodríguez-Ropero F.; Hajari T.; Van Der Vegt N. F. A. Mechanism of Polymer Collapse in Miscible Good Solvents. J. Phys. Chem. B 2015, 119 (51), 15780–15788. 10.1021/acs.jpcb.5b10684. 26619003

[ref6] Zhu P. W.; Napper D. H. Coil-to-Globule Type Transitions and Swelling of Poly(N-Isopropylacrylamide) and Poly(Acrylamide) at Latex Interfaces in Alcohol-Water Mixtures. J. Colloid Interface Sci. 1996, 177, 343–352. 10.1006/jcis.1996.0042.

[ref7] Puvvada S.; Blankschtein D. Molecular-Thermodynamic Approach to Predict Micellization, Phase Behavior and Phase Separation of Micellar Solutions. I. Application to Nonionic Surfactants. J. Chem. Phys. 1990, 92 (6), 3710–3724. 10.1063/1.457829.

[ref8] Srinivasan V.; Blankschtein D. Effect of Counterion Binding on Micellar Solution Behavior: 1. Molecular-Thermodynamic Theory of Micellization of Ionic Surfactants. Langmuir 2003, 19 (23), 9932–9945. 10.1021/la030069v.

[ref9] Srinivasan V.; Blankschtein D. Effect of Counterion Binding on Micellar Solution Behavior: 2. Prediction of Micellar Solution Properties of Ionic Surfactant-Electrolyte Systems. Langmuir 2003, 19 (23), 9946–9961. 10.1021/la030070u.

[ref10] Alexandridis P.; Olsson U.; Lindman B. Phase Behavior of Amphiphilic Block Copolymers in Water-Oil Mixtures: The Pluronic 25R4-Water-p-Xylene System. J. Phys. Chem. 1996, 100, 280 10.1021/jp951626s.

[ref11] Israelachvil J. N.; Mitchell D. J.; Ninham B. W. Theory of Self-Assembly of Hydrocarbon Amphiphiles into Micelles and Bilayers. J. Chem. Soc., Faraday Trans. 2 1976, 72, 1525 10.1039/F29767201525.

[ref12] Brupbacher J. M.; Kern R. D.; O’Grady B. V. Reaction of hydrogen and carbon dioxide behind reflected shock waves. J. Phys. Chem. 1976, 80, 1031 10.1021/j100551a001.

[ref13] Griffin W. C. Classification of Surface-Active Agents by “HLB”. J. Cosmet Sci. 1949, 1, 311–326.

[ref14] Davidson M. L.; Laufer L.; Gottlieb M.; Walker L. M. Transport of Flexible, Oil-Soluble Diblock and BAB Triblock Copolymers to Oil/Water Interfaces. Langmuir 2020, 36 (26), 7227–7235. 10.1021/acs.langmuir.0c00477. 32482075

[ref15] Punjabi S. H.; Sastry N. V.; Aswal V. K.; Goyal P. S. Effect of Surfactants on Association Characteristics of Di- And Triblock Copolymers of Oxyethylene and Oxybutylene in Aqueous Solutions: Dilute Solution Phase Diagrams, SANS, and Viscosity Measurements at Different Temperatures. Int. J. Polym. Sci. 2011, 2011, 1–13. 10.1155/2011/570149.

[ref16] Noolandi J. Multiblock Copolymers as Polymeric Surfactants: Are “Pancakes” Better than “Dumbbells”?. Macromol. Theory Simul. 1992, 1 (5), 295–298. 10.1002/mats.1992.040010503.

[ref17] Gotchev G.; Kolarov T.; Khristov K.; Exerowa D. Electrostatic and Steric Interactions in Oil-in-Water Emulsion Films from Pluronic Surfactants. Adv. Colloid Interface Sci. 2011, 168 (1–2), 79–84. 10.1016/j.cis.2011.05.001. 21616474

[ref18] Lucassen-Reynders E. H. A Surface Equation of State for Mixed Surfactant Monolayers. J. Colloid Interface Sci. 1972, 41, 156 10.1016/0021-9797(72)90098-7.

[ref19] Ruckenstein E.; Li B. Surface Equation of State for Insoluble Surfactant Monolayers at the Air/Water Interface. J. Phys. Chem. B 1998, 102, 981 10.1021/jp972748i.

[ref20] Llamas S.; Guzmán E.; Akanno A.; Fernández-Peña L.; Ortega F.; Campbell R. A.; Miller R.; Rubio R. G. Study of the Liquid/Vapor Interfacial Properties of Concentrated Polyelectrolyte-Surfactant Mixtures Using Surface Tensiometry and Neutron Reflectometry: Equilibrium, Adsorption Kinetics, and Dilational Rheology. J. Phys. Chem. C 2018, 122 (8), 4419–4427. 10.1021/acs.jpcc.7b12457.

[ref21] Penfold J.; Thomas R. K. K. The application of the specular reflection of neutrons to the study of surfaces and interfaces. J. Phys.: Condens. Matter 1990, 2, 1369 10.1088/0953-8984/2/6/001.

[ref22] Zhang J.; Taylor D. J. F.; Li P. X.; Thomas R. K.; Wang J. B.; Penfold J. B. Adsorption of DNA and Dodecyl Trimethylammonium Bromide Mixtures at the Air/Water Interface: A Neutron Reflectometry Study. Langmuir 2008, 24 (5), 1863–1872. 10.1021/la7021566. 18220428

[ref23] Fainerman V. B.; Miller R.; Ferri J. K.; Watzke H.; Leser M. E.; Michel M. Reversibility and Irreversibility of Adsorption of Surfactants and Proteins at Liquid Interfaces. Adv. Colloid Interface Sci. 2006, 123–126, 163–171. 10.1016/j.cis.2006.05.023. 16843423

[ref24] Rosales A. M.; Segalman R. A.; Zuckermann R. N. Polypeptoids: A Model System to Study the Effect of Monomer Sequence on Polymer Properties and Self-Assembly. Soft Matter 2013, 9 (35), 8400–8414. 10.1039/c3sm51421h.

[ref25] Rosales A. M.; Murnen H. K.; Kline S. R.; Zuckermann R. N.; Segalman R. A. Determination of the Persistence Length of Helical and Non-Helical Polypeptoids in Solution. Soft Matter 2012, 8 (13), 3673–3680. 10.1039/c2sm07092h.

[ref26] Murnen H. K.; Khokhlov A. R.; Khalatur P. G.; Segalman R. A.; Zuckermann R. N. Impact of Hydrophobic Sequence Patterning on the Coil-to-Globule Transition of Protein-like Polymers. Macromolecules 2012, 45 (12), 5229–5236. 10.1021/ma300707t.

[ref27] Patterson A. L.; Danielsen S. P. O.; Yu B.; Davidson E. C.; Fredrickson G. H.; Segalman R. A. Sequence Effects on Block Copolymer Self-Assembly through Tuning Chain Conformation and Segregation Strength Utilizing Sequence-Defined Polypeptoids. Macromolecules 2019, 52 (3), 1277–1286. 10.1021/acs.macromol.8b02298.

[ref28] Zhang D.; Lahasky S. H.; Guo L.; Lee C. U.; Lavan M. Polypeptoid Materials: Current Status and Future Perspectives. Macromolecules 2012, 45, 5833–5841. 10.1021/ma202319g.

[ref29] Chan B. A.; Xuan S.; Li A.; Simpson J. M.; Sternhagen G. L.; Yu T.; Darvish O. A.; Jiang N.; Zhang D. Polypeptoid Polymers: Synthesis, Characterization, and Properties. Biopolymers 2018, 109 (1), e23070 10.1002/bip.23070. 29068055

[ref30] Zuckermann R. N.; Kerr J. M.; Kent S. B. H.; Moos W. H. Efficient Method for the Preparation of Peptoids [Oligo(N-Substituted Glycines)] by Submonomer Solid-Phase Synthesis. J. Am. Chem. Soc. 1992, 114 (26), 10646–10647. 10.1021/ja00052a076.

[ref31] Connolly M. D.; Xuan S.; Molchanova N.; Zuckermann R. N. Submonomer Synthesis of Sequence Defined Peptoids with Diverse Side-Chains. Methods in Enzymology; Academic Press Inc., 2021; Vol. 656, pp 241–270. 34325788 10.1016/bs.mie.2021.04.022

[ref32] Tahery R.; Modarress H.; Satherley J. Density and Surface Tension of Binary Mixtures of Acetonitrile + 1-Alkanol at 293.15 K. J. Chem. Eng. Data 2006, 51 (3), 1039–1042. 10.1021/je050519g.

[ref33] Reichert M. D.; Walker L. M. Interfacial Tension Dynamics, Interfacial Mechanics, and Response to Rapid Dilution of Bulk Surfactant of a Model Oil-Water-Dispersant System. Langmuir 2013, 29 (6), 1857–1867. 10.1021/la4000395. 23311916

[ref34] Alvarez N. J.; Walker L. M.; Anna S. L. A Microtensiometer to Probe the Effect of Radius of Curvature on Surfactant Transport to a Spherical Interface. Langmuir 2010, 26 (16), 13310–13319. 10.1021/la101870m. 20695573

[ref35] Alvarez N. J.; Vogus D. R.; Walker L. M.; Anna S. L. Using Bulk Convection in a Microtensiometer to Approach Kinetic-Limited Surfactant Dynamics at Fluid-Fluid Interfaces. J. Colloid Interface Sci. 2012, 372 (1), 183–191. 10.1016/j.jcis.2011.12.034. 22326047

[ref36] Kirby S. M.; Anna S. L.; Walker L. M. Sequential Adsorption of an Irreversibly Adsorbed Nonionic Surfactant and an Anionic Surfactant at an Oil/Aqueous Interface. Langmuir 2015, 31 (14), 4063–4071. 10.1021/la504969v. 25798716

[ref37] Hodges C. S.; Biggs S.; Walker L. Complex Adsorption Behavior of Rodlike Polyelectrolyte - Surfactant Aggregates. Langmuir 2009, 25 (8), 4484–4489. 10.1021/la8033534. 19260656

[ref38] Reichert M. D.; Walker L. M. Coalescence Behavior of Oil Droplets Coated in Irreversibly-Adsorbed Surfactant Layers. J. Colloid Interface Sci. 2015, 449, 480–487. 10.1016/j.jcis.2015.02.032. 25766654

[ref39] Davidson M. L.; Walker L. M. Interfacial Properties of Polyelectrolyte-Surfactant Aggregates at Air/Water Interfaces. Langmuir 2018, 34 (43), 12906–12913. 10.1021/acs.langmuir.8b02438. 30274519

[ref40] Tein Y. S.; Thompson B. R.; Majkrzak C.; Maranville B.; Renggli D.; Vermant J.; Wagner N. J. Instrument for Measurement of Interfacial Structure-Property Relationships with Decoupled Interfacial Shear and Dilatational Flow: ″Quadrotrough ″. Rev. Sci. Instrum. 2022, 93 (9), 093903 10.1063/5.0090350. 36182507

[ref41] Lucassen-Reynders E. H.; Cagna A.; Lucassen J. Gibbs Elasticity, Surface Dilational Modulus and Diffusional Relaxation in Nonionic Surfactant Monolayers. Colloids Surf., A 2001, 186, 63–72. 10.1016/s0927-7757(01)00483-6.

[ref42] Lucassen J.; Van Den Tempel M. Dynamic Measurements of Dilational Properties of a Liquid Interface. Chem. Eng. Sci. 1972, 27, 1283–1291. 10.1016/0009-2509(72)80104-0.

[ref43] Joos P. Dynamic Surface Phenomena; CRC Press: The Netherlands, 1999.

[ref44] Kotula A. P.; Anna S. L. Regular Perturbation Analysis of Small Amplitude Oscillatory Dilatation of an Interface in a Capillary Pressure Tensiometer. J. Rheol 2015, 59 (1), 85–117. 10.1122/1.4902546.

[ref45] Scriven L. E. Dynamics of a Fluid Interface Equation of Motion for Newtonian Surface Fluids. Chem. Eng. Sci. 1960, 12, 98–108. 10.1016/0009-2509(60)87003-0.

[ref46] Hunter C. A.; Sanders J. K. M. The nature of .pi.-.pi. interactions. J. Am. Chem. Soc. 1990, 112 (14), 5525–5534. 10.1021/ja00170a016.

[ref47] Evers O. A.; Scheutjens J. M. H. M.; Fleer G. J. Statistical Thermodynamics of Block Copolymer Adsorption Part 2.-Effect of Chain Composition on the Adsorbed Amount and Layer Thickness. J. Chem. Soc., Faraday Trans. 1990, 86, 1333–1340. 10.1039/ft9908601333.

[ref48] Siqueira D. F.; Stamm M.; Breiner U.; Stadler R. Adsorption of Di-and Triblock Copolymers with Functionalized Butadiene-Styrene Blocks from Dilute Solution. Polymer 1995, 36, 3229–3233. 10.1016/0032-3861(95)97887-l.

