# Supplementary material for: Polypeptoid Monomer Sequence and Chemical Composition as Independent Controls of Interfacial Tension and Elasticity at Air/Fluid Interfaces
Source: Langmuir. 2024 Jul 12;40(29):15353–62. doi: 10.1021/acs.langmuir.4c02195 (PMC11270983; doi:10.1021/acs.langmuir.4c02195)
Supplement: Supplementary file 1 — la4c02195_si_001.pdf [file la4c02195_si_001.pdf]

Supporting Information - Polypeptoid monomer sequence and chemical composition as independent controls of interfacial tension and elasticity at air/fluid interfaces

Michal Roguski[1†], Michael L. Davidson[1†], Audra J. DeStefano[2], Rachel A. Segalman[2,3], and Lynn M. Walker*[4]

[1] Department of Chemical Engineering, Carnegie Mellon University, Pittsburgh, PA, 15213

[2] Department of Chemical Engineering, University of California, Santa Barbara, CA, 93106

[3] Department of Materials, University of California, Santa Barbara, CA, 93106

[4] Department of Chemical Engineering & Materials Science, University of Minnesota, Minneapolis, MN, 55455

[†] shared first authorship, these authors contributed equally.

*corresponding author: lmwalker@umn.edu

Details:

This supporting information contains 2 pages and 1 figure.

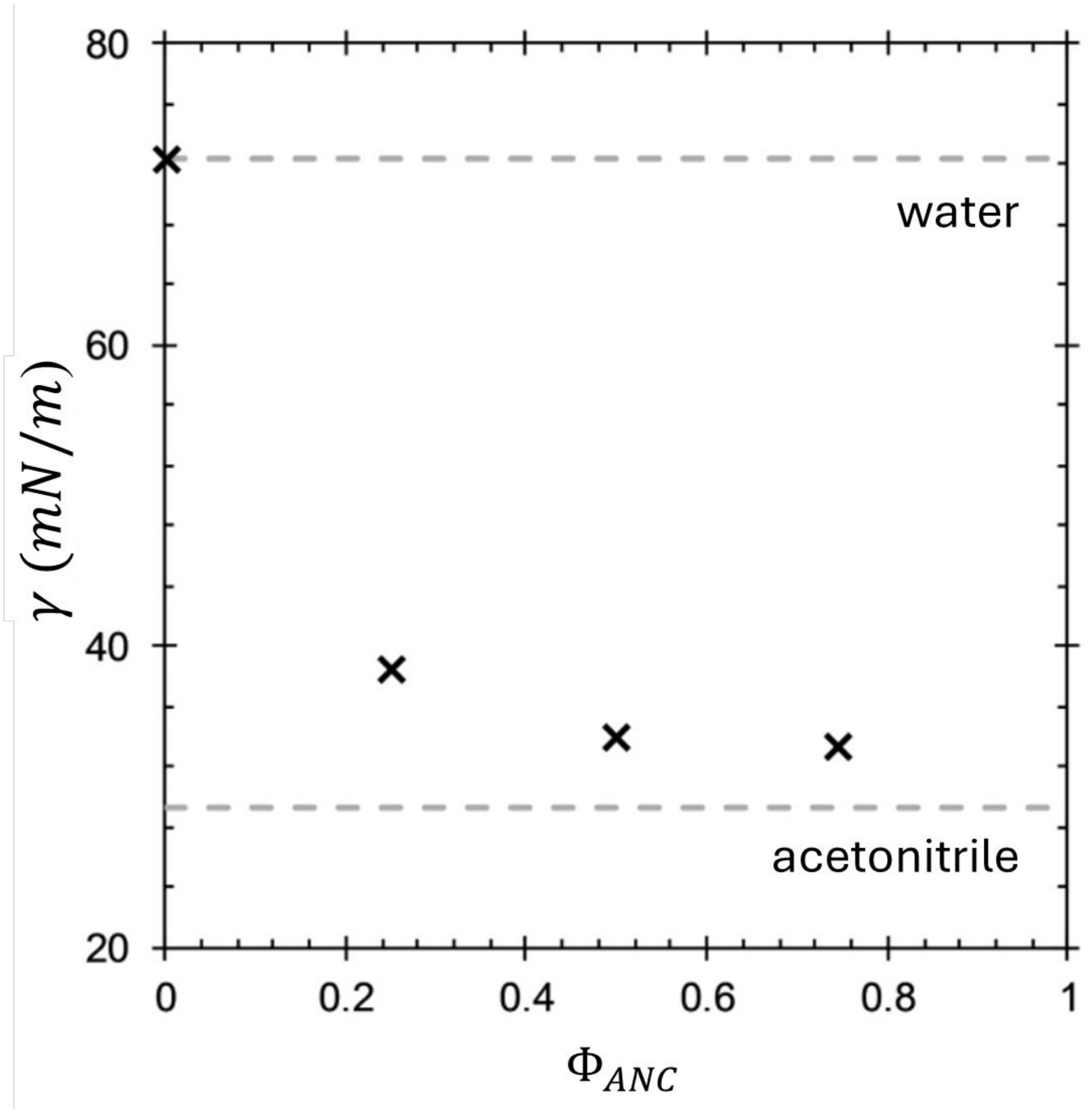

Figure S1. Surface tension of acetonitrile/water mixtures measured with the microtensiometer. Dashed lines are the surface tensions of the pure solvents.[1]

Figure S1 shows the results of measurements of the interfacial tension between air and mixtures of acetonitrile and water of different volume fractions of acetonitrile (ϕ_{ACN}). The dashed lines show values of the interfacial tension of pure components (72.2 mN/m for pure water, and 29.3 mN/m for pure acetonitrile).

References:

1. Tahery, R.; Modarress, H.; Satherley, J. J. Chem. Eng. Data 2006, 51 (3), 1039–1042
